# Circulating Cell-Free DNA Analysis for Diagnostic and Prognostic Assessment of Hepatocellular Carcinoma in Cirrhosis

**DOI:** 10.3390/ijms27125590

**Published:** 2026-06-20

**Authors:** Inés Aznar-Peralta, Amparo Roa-Colomo, Javier López Hidalgo, Cristobal Fresno, Valeria Denninghoff, María José Serrano

**Affiliations:** 1 GENYO, Centre for Genomics and Oncological Research, Pfizer/University of Granada/Andalusian Regional Government, 18016 Granada, Spain; ines.aznar@genyo.es; 2 Digestive System Unit, Costa del Sol University Hospital, 29603 Marbella, Spain; amparo.roa.colomo@gmail.com; 3 Department of Pathology, San Cecilio Clinical University Hospital—Instituto de Investigación Biosanitaria ibs GRANADA, 18012 Granada, Spain; javierl.lopez.sspa@juntadeandalucia.es; 4 Department of Pathology, Virgen de las Nieves University Hospital—Instituto de Investigación Biosanitaria ibs GRANADA, 18012 Granada, Spain; 5 Research Support Network, Scientific Research Coordination, National Autonomous University of Mexico, Mexico City 04510, Mexico; cristobalfresno@gmail.com; 6 Molecular-Clinical Laboratory, University of Buenos Aires (UBA)—National Council for Scientific and Technical Research (CONICET), Buenos Aires C1053, Argentina

**Keywords:** circulating free DNA, fragmentomics, cancer biomarker, hepatocarcinoma, cirrhosis, liquid biopsy, prognostic and predictive markers

## Abstract

Early detection of hepatocellular carcinoma (HCC) is crucial for curative treatment, yet current screening strategies for high-risk liver cirrhosis (LC) patients lack sufficient sensitivity. This study evaluates plasma cell-free DNA(cfDNA) concentration and fragmentomics as biomarkers to improve HCC diagnosis and prognosis. Plasma samples from 39 HCC and 46 LC patients were analyzed for cfDNA concentration and fragment patterns. A multivariate logistic regression model (CMAC), integrating cfDNA concentration, mononucleosome proportion (%MN), alpha-fetoprotein (AFP), and c-reactive protein (CRP), was developed and validated using Leave-One-Out Cross-Validation and bootstrapping. HCC patients exhibited significantly higher cfDNA concentrations (*p* < 0.0001) and longer fragment lengths (*p* < 0.05) compared to LC patients. The CMAC model demonstrated superior diagnostic performance (AUROC = 0.946) compared to AFP alone (AUROC = 0.777, *p* < 0.001). Notably, in early-stage HCC, the CMAC model remained highly accurate (AUROC = 0.941), whereas AFP failed to reach statistical significance. Higher CMAC scores were significantly associated with advanced BCLC stages (*p* = 0.009), lymphovascular invasion (*p* = 0.0063) and reduced overall survival (*p* = 0.0037). Integration of cfDNA analysis with established clinical markers in the CMAC model shows promise as a complementary tool for the early detection of HCC in LC patients. Validation in larger, multicenter cohorts will be necessary to confirm these findings and their clinical applicability.

## 1. Introduction

Liver cancer represents a significant global health challenge, ranking as the sixth most common cancer worldwide and third leading cause of cancer-related deaths [1]. Projections suggest that, by 2040, 1.3 million individuals will be affected by liver cancer annually [2].

Hepatocellular carcinoma (HCC) represents approximately 90% of all primary liver cancers, characterized by its aggressive nature and poor prognosis when diagnosed at an advanced stage. Patients diagnosed at early stages have access to potentially curative treatments such as surgical resection, liver transplantation, or radiofrequency ablation, significantly enhancing their long-term survival prospects [3]. Unfortunately, more than 50% of HCC cases are diagnosed at late stages or incidentally, severely limiting curative options with dramatically worse prognosis [4]. This delayed diagnosis is primarily attributed to the asymptomatic nature of early-stage HCC, lack of awareness, insufficient screening programs, and limited sensitivity or accessibility of current diagnostic approaches [5,6].

HCC typically develops in the setting of chronic liver disease, with cirrhosis being the strongest risk factor [7,8]. Over 90% of HCC cases occur in patients with underlying chronic liver disease, and liver cirrhosis (LC) of any etiology significantly increases the risk of HCC development [3,9]. Given the strong association between LC and HCC, surveillance programs have been implemented for this at-risk population. Current guidelines recommend semiannual screening for HCC in cirrhotic patients using abdominal ultrasound. The combined use of ultrasound with alpha fetoprotein (AFP) increases sensitivity, making it a feasible option [7]. However, these methods have limitations in terms of sensitivity and specificity, especially for early-stage disease detection [4,8].

Liquid biopsy, particularly the analysis of circulating cell-free DNA (cfDNA), has shown promise for detecting HCC at earlier stages [10,11]. This noninvasive approach offers several advantages over traditional screening methods, including the potential for more frequent monitoring and the ability to capture tumor heterogeneity, but more importantly, increased sensitivity. cfDNA can refer to fragments of DNA detected in both healthy individuals and patients with cancer that are derived from necrotic or apoptotic cells, or actively released by living cells [12,13]. Research interest has been focused on the molecular characteristics of cfDNA in HCC, including methylation patterns [11,14] and hotspot mutations [15]. Fragmentomics has emerged as a new research field that focuses on cfDNA size distribution and fragmentation patterns [13,16].

This observational cross-sectional study aimed to address the urgent need for improved HCC detection methods by investigating cfDNA analysis as a novel approach to differentiate between LC and HCC.

## 2. Results

### 2.1. Clinicopathological Characteristics of Study Cohort

**Figure 1:**
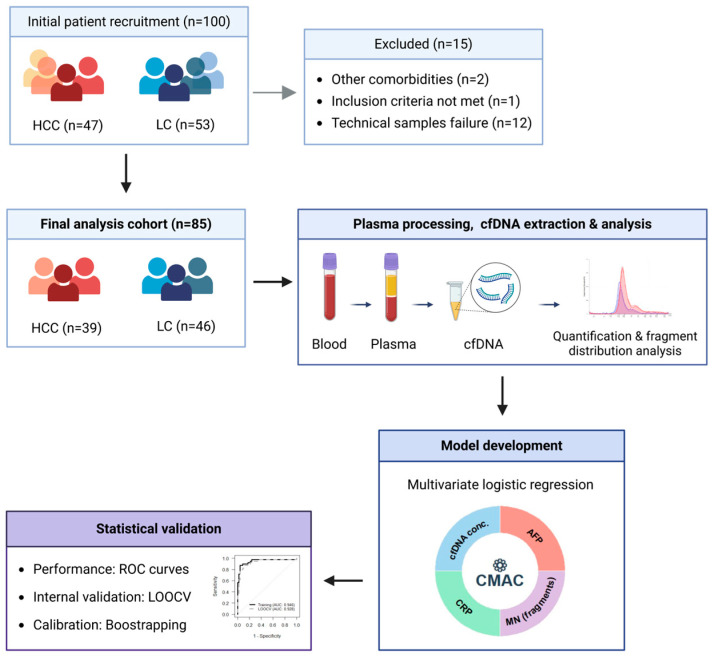
Study flowchart and analytical pipeline. Of the 100 patients initially screened, 15 were excluded due to other comorbidities (*n* = 2), failure to meet inclusion criteria (*n* = 1), or technical sample failure (*n*= 12). The final analytical cohort comprised 85 patients, categorized into HCC (*n* = 39) and LC (*n* = 46). Following plasma processing and cfDNA extraction, a predictive model for HCC detection (CMAC Score) was constructed using multivariate logistic regression including cfDNA, CRP, AFP, and % MN. The model was rigorously validated through internal cross-validation via LOOCV, calibration via bootstrapping (1000 repetitions), and performance assessment via ROC curves and DeLong tests. Abbreviations: AFP, alpha-fetoprotein; cfDNA, cell-free DNA; CMAC, concentration, mononucleosome, alpha-fetoprotein and C-reactive protein; CRP, C-reactive protein; HCC, hepatocellular carcinoma; LC, liver cirrhosis; LOOCV, Leave-One-Out Cross-Validation; MN, mononucleosome; ROC, Receiver Operating Characteristic. Created in BioRender.Inés Aznar-Peralta. (2026) https://BioRender.com.

Our study included a total of 85 individuals, 46 cancer-free LC patients and 39 HCC patients, all of whom also had underlying cirrhosis. The study flowchart is shown in Figure 1, and a descriptive analysis of the cohort is presented in Table 1. The cancer cohort was subdivided by stage into early stage (ES), comprising 38.5% of the group, and advanced stage (AS), comprising 61.5%. Supplementary Table S1 presents a comprehensive overview of the characteristics of the newly defined cohorts. Treatment of HCC was classified as surgical treatment (with curative intent) or no surgical treatment, with most patients (76.9%) receiving the latter. Lymphovascular invasion (LVI) was detected in 12.8% of cancer patients.

The Mann–Whitney test revealed differences (*p* < 0.05, Table 1) in several clinical parameters between LC and HCC cohorts: levels of the tumor biomarker AFP, the acute-phase reactant C-reactive protein (CRP), and liver function parameters aspartate transaminase (AST), gamma-glutamyltransferase (GGT) and alkaline phosphatase (ALP) were significantly higher in HCC patients, while albumin was lower, indicating impaired liver function and liver damage, characteristic of HCC.

### 2.2. Differential cfDNA Concentration and Fragmentation Patterns in HCC

**Figure 2:**
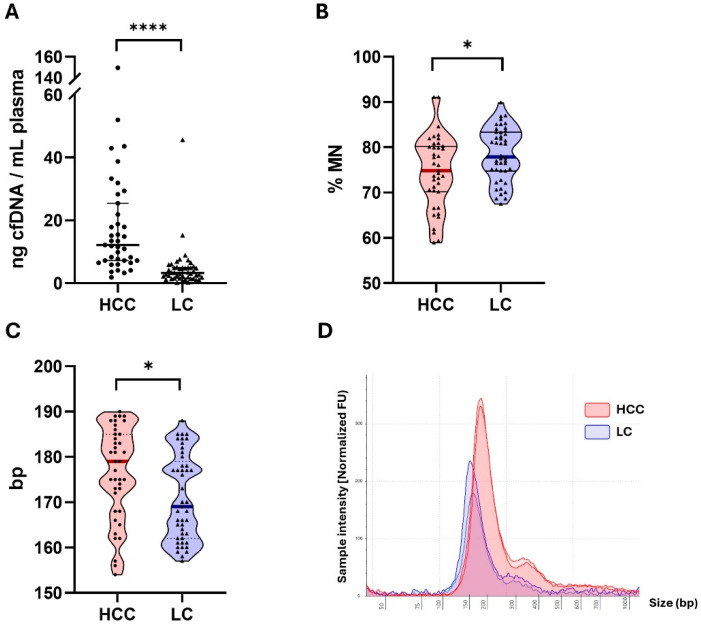
cfDNA characteristics in HCC and LC groups. Differences between HCC and LC groups in (**A**) cfDNA concentration, (**B**) MN proportion and (**C**) mean size of MNs in bp. (**D**) Representation of cfDNA fragments’ profile of HCC patients (red) and LC patients (blue). *p*-values for Mann–Whitney test: **** *p* < 0.0001; * *p* < 0.05. Abbreviations: cfDNA, circulating cell-free DNA; LC, liver cirrhosis; MN, mononucleosome.

Concentration of cfDNA in plasma ranged from 0.24 ng/mL to 149.5 ng/mL across the entire cohort. HCC patients exhibited significantly higher (*p* < 0.0001) median cfDNA concentrations (12.15 ng/mL, IQR: 7.24–25.41) compared to cirrhotic individuals (3.26 ng/mL, IQR: 1.60–5.04) (Figure 2A). Fragment size distribution was studied by comparing two variables: the percentage of mononucleosomes (% MN), which include those of size 100–250 bp, and the mean size of these fragments, represented in bp. HCC samples showed a significantly lower (*p* < 0.05) median MN proportion (74.85%, IQR: 70.19–80.21) compared to LC samples (77.88%, IQR: 74.79–83.35) (Figure 2B). Furthermore, the mean length of these fragments was significantly greater (*p* < 0.05) in the HCC group (179 bp, IQR: 168.0–185.0) than in the LC group (169.0 bp, IQR: 162.0–179.0) (Figure 2C). The fragmentomic profiles visualized by capillary electrophoresis revealed a distinct rightward shift in the HCC (red) peaks, alongside a more prominent dinucleosome peak compared to the LC (blue) profiles. These results indicate a lower proportion of short MN fragments and an overall shift toward higher molecular weight fragments in the presence of malignancy (Figure 2D).

### 2.3. Development of CMAC Model for Distinguishing HCC from LC

Multivariate backward stepwise binary logistic regression analysis was conducted to identify independent predictors of HCC and to construct a robust diagnostic probability model. The final model included four variables that significantly contributed to the diagnosis, cfDNA concentration (OR = 1.105, 95%CI = 1.015–1.203, *p* = 0.021), % MN (OR = 0.912, 95%CI = 0.838–0.993, *p* = 0.034), AFP (OR = 1.347, 95%CI = 1.057–1.716, *p* = 0.016) and CRP (OR = 1.194, 95%CI = 1.033–1.380, *p* = 0.017), and was therefore named CMAC (concentration, MN, AFP and CRP). The CMAC model demonstrated high calibration accuracy and robust goodness-of-fit, as evidenced by the Hosmer–Lemeshow test (*p*-value = 0.461). CMAC is defined as follows:

CMAC = 2.829 + 0.1 × [ng cfDNA/mL plasma] − 0.092 × [% MN] + 0.298 × [AFP (ng/mL)] + 0.177 * [CRP (mg L)]

**Figure 3:**
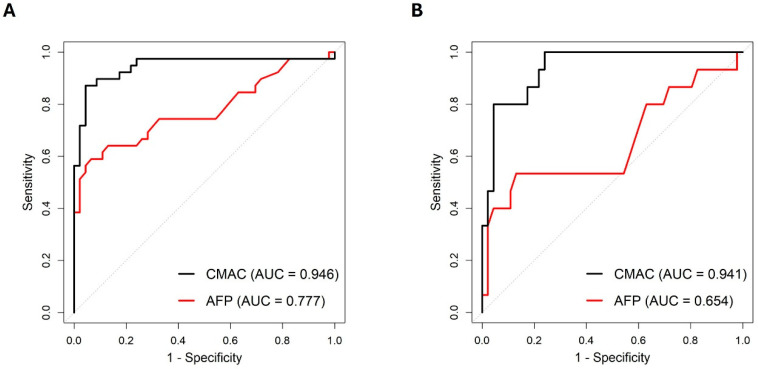
Comparison of diagnostic performance between the CMAC model and AFP. ROC curves comparing the diagnostic accuracy of the CMAC score (black line) and AFP (red line) in (**A**) the total study cohort and (**B**) the ES HCC subgroup. Statistical significance for the difference in AUC between CMAC and AFP was determined by DeLong’s test. Diagonal dashed line represents the line of identity (AUC = 0.5). Abbreviations: AFP, alpha-fetoprotein; AUC, area under the curve; CMAC, concentration, mononucleosome, alpha-fetoprotein and C-reactive protein.

The probabilities of HCC calculated with the CMAC model (the CMAC score) demonstrated remarkable diagnostic performance in discriminating HCC from patients with liver cirrhosis (LC), achieving an area under the Receiver Operating Characteristic (AUROC) of 0.946 (95% CI = 0.890–1.000, *p* < 0.001), outperforming its individual components (Figure S1). Importantly, the model’s performance was significantly (*p* < 0.001) higher than that of AFP alone, which yielded an AUROC of 0.777 (95% CI = 0.672–0.882, *p* < 0.001) (Figure 3A), confirming its robust incremental value for HCC detection. At the optimal threshold of 0.425, the model achieved a sensitivity of 87.2% (95% CI= 51.3–94.9%) and a specificity of 95.7% (95% CI= 73.9–100%). Internal validation via Leave-One-Out Cross-Validation (LOOCV) confirmed the model’s stability, yielding a cross-validated AUROC of 0.928, with a sensitivity of 79.5% and a specificity of 93.5% (Figure S2). Furthermore, the model exhibited excellent calibration, with a Brier score of 0.101 and a mean absolute prediction error of 0.035 based on 1000 bootstrap resamples (Figure S3), indicating a high degree of agreement between predicted probabilities and observed HCC outcomes.

The CMAC model demonstrated significant associations with various clinical and pathological characteristics (Table 2). Higher scores were notably associated with advanced BCLC stages (*p* = 0.009) and the presence of LVI (*p* = 0.0063) (Figure S4), demonstrating a strong correlation between the model’s score and tumor aggressiveness. As expected, the score was strongly correlated with its constituent components, CRP (*p* < 0.001) and AFP (*p*< 0.001), confirming the internal consistency of the model. Furthermore, the calculated score showed significant correlations with independent markers of liver dysfunction and biliary stress, such as GGT (*p* < 0.001) and albumin (*p* < 0.001), as well as alterations in coagulation parameters like the international normalized ratio (INR) (*p* = 0.003) (Figure S5). These findings suggest that the CMAC model effectively integrates inflammatory and tumoral signals to reflect both the biological aggressiveness of the disease and the underlying hepatic impairment.

### 2.4. Performance of CMAC Score for Early HCC Detection

To further assess the clinical utility of the model in early diagnosis, the CMAC score was evaluated in the subgroup of patients with ES HCC (*n* = 15). The score maintained an outstanding performance with an AUROC of 0.941 (95% CI = 0.883–1.000, *p* < 0.001) (Figure 3B), demonstrating that the diagnostic signal remains robust even in patients with low tumor burden. The optimal diagnostic threshold remained consistent at 0.425, and at this cutoff, the model yielded a sensitivity of 80% and a remarkable specificity of 95.7%. In contrast, the conventional biomarker AFP showed a statistically non-significant diagnostic performance in this early-stage setting, with an AUROC of 0.654 (95% CI= 0.471–0.837, *p* = 0.076). When comparing both performances, the CMAC score significantly outperformed AFP (*p* = 0.002) (Figure 3B). These findings highlight that while AFP fails to reliably distinguish between patients and controls at curative stages, the CMAC score provides a robust and superior alternative for early HCC detection.

### 2.5. Prognosis Factors in HCC Patients

**Figure 4:**
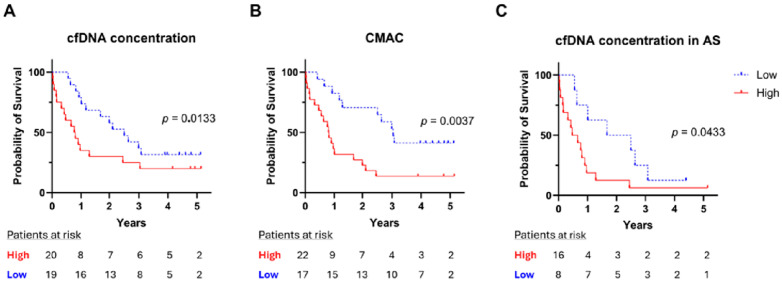
Kaplan–Meier survival analysis of HCC patients. Overall survival for the entire HCC cohort based on (**A**) high or low cfDNA concentration, with a cutoff of 12.0 ng cfDNA/mL plasma, and (**B**) CMAC score, with a cutoff of 0.953. (**C**) Survival analysis of AS HCC patients stratified by high or low cfDNA concentration, with a cutoff of 12.0 ng cfDNA/mL plasma. Cutoff values were determined using 1-year survival ROC curves and the Youden Score for the studied variable in the population of interest. *p*-values were calculated using the log-rank test. Abbreviations: AS, advanced stage; cfDNA, cell-free DNA; CMAC, concentration, mononucleosome, alpha-fetoprotein and C-reactive protein.

During the 5-year follow-up period of this study, mortality in the HCC group reached 74.36% (Table 1), with median overall survival (OS) of 10.8 months. Concentration was the only cfDNA parameter associated with worse OS in Kaplan–Meier analysis (*p* = 0.0133, Figure 4A). Specifically, the median OS of patients with high cfDNA levels was 9.6 months, in contrast to 27.5 among patients with low concentration.

Univariate Cox’s proportional hazard regression analyses showed that, in addition to cfDNA concentration, several clinical variables were associated with worse prognosis for HCC patients: BCLC stage, treatment, and levels of AFP, CRP and GGT (Table 3). Multivariable analysis identified three significant factors associated with OS: early or advanced BCLC stage (HR = 4.049, 95%CI: 1.675–9.788, *p* = 0.002), cfDNA concentration (HR = 1.027, 95%CI = 1.007–1.047), *p* = 0.008), and GGT levels (HR = 1.003, 95%CI = 1.000–1.005, *p* = 0.018) (Table 3).

The previously calculated CMAC score also correlated with HCC patient survival, revealed not only by univariate Cox regression (Table 3) but also by Kaplan–Meier analysis (Figure 4B). HCC patients with a higher CMAC score had a median OS of 9.7 months, while the median OS for those with a lower score was 36.1 months.

Given that BCLC stage was the most significant variable associated with patient prognosis in the Cox regression analysis, we also studied OS for early (ES, *N* = 15) and advanced stage (AS, *N* = 24) HCC separately (Table 3). We found no variable indicative of prognosis in early-stage HCC patients; however, cfDNA concentration in plasma was identified by Cox regression as the only independent prognostic factor correlated with shorter OS in advanced-stage HCC patients (HR= 1.031, 95%CI = 1.010–1.052, *p* = 0.004), as also seen with Kaplan–Meier analysis (*p* = 0.043, Figure 4C).

## 3. Discussion

HCC continues to pose a significant global health challenge, with early detection playing a pivotal role in improving patients’ outcomes. Despite breakthroughs in systemic therapies for advanced-stage patients, such as the combination of atezolizumab and bevacizumab [17], the focus must remain on early diagnosis as it offers the best opportunity for curative treatments such as liver transplantation or resection.

Current screening strategies, primarily based on alpha-fetoprotein (AFP), frequently lack sufficient sensitivity. In our cohort, AFP levels in HCC patients ranged from 3.2 to 42.6 ng/mL (median 7.65 ng/mL), values notably lower than the 20 ng/mL threshold established by clinical guidelines [7]. In fact, 67.7% (26/39) of the patients had AFP levels below this clinical cutoff. Moreover, the AUROC for AFP was only 0.777, reflecting its low performance in discriminating between LC and HCC. Critically, when focusing on ES HCC, the ROC analysis for AFP failed to reach statistical significance (*p* > 0.05), rendering it ineffective as a standalone diagnostic tool for the patients who would benefit most from curative interventions. This is consistent with other studies reporting AFP’s limited diagnostic performance even when the threshold is lowered [18,19,20].

In this context, there is an urgent need for novel biomarkers that are simple to analyze, rapid, and cost-effective. To address this gap, we evaluated cfDNA as a diagnostic and prognostic tool in HCC. The results demonstrated that plasma cfDNA concentration was significantly higher in patients with HCC, with levels approximately four times higher than those observed in LC individuals without HCC. These findings are in line with previous reports supporting cfDNA quantification as a biomarker of disease burden [21,22,23,24].

Beyond concentration, cfDNA fragmentation profiles provided valuable differential information between LC and HCC. Cirrhotic patients predominantly exhibited shorter, apoptotic nucleosome fragments, whereas HCC patients were characterized by longer and more heterogeneous fragments, consistent with necrosis and chromatin disorganization [25]. This may reflect the influence of tumor origin on cfDNA size distribution and fragmentation patterns [13,16].

We integrated cfDNA concentration, MN proportion, AFP, and CRP into a composite diagnostic model (CMAC). This approach achieved high diagnostic accuracy for distinguishing HCC from LC, outperforming each variable individually and notably improving AFP alone. By combining the specificity of conventional markers like AFP and CRP with the biological sensitivity of cfDNA fragmentomics, the CMAC model provides a more comprehensive molecular snapshot of the disease. The integration of these parameters, each of which may carry inherent non-specificities when used in isolation, allows the CMAC model to maintain high diagnostic performance by balancing their individual limitations through a multiparametric approach. This strategy is consistent with approaches proposed in recent studies [18,19] and highlights the advantage of combining cfDNA with traditional clinical markers.

To further ensure the reliability of these findings, the model was subjected to rigorous internal validation. The stability of the CMAC score was confirmed through both LOOCV and 1000 bootstrap resamples. Both methods consistently yielded high AUROC values and excellent calibration, demonstrating that the model’s accuracy is not a result of overfitting but a reflection of its robust generalizability. This high degree of agreement between predicted and observed outcomes, even after intensive resampling, reinforces the potential of the CMAC model as a trustworthy clinical tool.

Our analysis revealed significant associations between the CMAC model and several established markers of liver function and damage in the entire cohort. We found positive correlations between the CMAC score and AST, GGT, and ALP, as well as a significant inverse correlation with albumin level. Within the HCC group, the CMAC score was also associated with advanced BCLC stage and LVI, both established prognostic factors in HCC. This relationship likely reflects increased tumor burden and more aggressive tumor biology, in line with prior studies linking high cfDNA levels with unfavorable outcomes [26].

Despite the smaller sample size of the ES HCC subgroup (*n* = 15), the CMAC model demonstrated remarkable diagnostic performance in these patients, effectively identifying cases that were otherwise missed by conventional biomarkers. While AFP failed to reach statistical significance in the ES cohort, our multiparametric score remained robust, suggesting its potential utility in the most critical clinical setting: the detection of tumors amenable to curative treatment.

Regarding the prognostic value of cfDNA, we discovered that higher cfDNA concentration was not only associated with shorter overall in the HCC cohort but was also the only independent prognostic factor in advanced-stage HCC patients. These findings support those of previous studies underscoring the potential utility of cfDNA in guiding clinical decisions and predicting outcomes [24,26]. Furthermore, the association between CMAC score and patient survival suggests its potential dual role in both diagnosis and prognosis, offering a comprehensive tool for clinicians.

Despite the promising findings, our study has limitations: a small sample size, particularly for early-stage HCC, and lack of external validation in a larger, multi-center independent cohort.. Specifically, our cohort features an imbalance in sex distribution (22 females vs. 63 males), which reflects the known male predominance in HCC epidemiology. While recent literature has reported that baseline cfDNA levels can exhibit significant sex-related differences [27], a sub-analysis within our specific study population revealed that the slightly higher cfDNA concentrations observed in male patients did not achieve statistical significance (*p* > 0.05). Nevertheless, this demographic imbalance remains a limitation, and future large-scale, sex-matched cohorts will be essential to definitively validate the generalizability of our model.

Additionally, we acknowledge that our fragmentomics analysis was targeted, focusing on the MN proportion as a global surrogate of chromatin structure rather than employing high-resolution genome-wide sequencing to assess end-motifs or specific size distributions. While advanced sequencing provides a deeper biological footprint, our approach offers a highly cost-effective and easily translatable alternative for routine clinical chemistry. Although the analytical workflow is feasible in clinical practice, prospective studies are needed to evaluate its cost-effectiveness and utility in real-world surveillance programs.

In conclusion, the CMAC model, integrating cfDNA characteristics with traditional biomarkers, could significantly enhance diagnostic precision for HCC and serve as a valuable prognostic indicator, offering a robust tool for clinical decision-making.

## 4. Materials and Methods

### 4.1. Patient Population

A total of 100 individuals were initially screened, of whom 85 met the eligibility criteria and were enrolled in this study. The final cohort consisted of 39 patients with HCC and 46 patients with LC without evidence of malignancy. Study inclusion criteria were defined as follows: (i) for the cirrhotic group, a confirmed diagnosis of Child-Pugh A cirrhosis without evidence of HCC, under active surveillance within a screening program; and (ii) for the HCC group, a diagnosis of treatment-naive HCC across any BCLC stage. The presence of vascular invasion was assessed via imaging criteria.

All participants were aged 18 years or older and provided written informed consent prior to blood collection. Recruitment took place at the time of diagnosis between 2018 and 2021 at the Gastroenterology and Hepatology Unit of San Cecilio University Hospital (Granada, Spain). The study protocol was approved by the Institutional Ethics Committee, adhering to the ethical guidelines of the Declarations of Helsinki and Istanbul. Detailed clinical and pathological characteristics of the study population are summarized in Table 1. For further analysis, the HCC cohort was stratified into two groups: ES, comprising BCLC stage A; and AS, comprising BCLC stages B, C, and D. Of note, no patients in BCLC stage 0 were identified or included in this study. Comprehensive clinical data for these subgroups are provided in Supplementary Table S1.

### 4.2. Laboratory Tests

Baseline hematological and biochemical parameters were retrieved from the clinical database, including platelet count, prothrombin activity, AFP, CRP, AST, ALP, GGT, albumin and alanine aminotransferase (ALT). Clinical data with a 5-year follow-up period were also included.

### 4.3. Blood Sample Collection and Plasma Extraction

Peripheral blood samples (10 mL) were collected in K2-EDTA Vacutainer tubes at the time of diagnosis. Blood samples were centrifuged at 2000× *g* for 10 min within 4 h of collection to isolate plasma. To ensure the complete removal of cellular debris, plasma supernatants were transferred to new microtubes and subjected to a second centrifugation at 16,000× *g* for 10 min. Plasma aliquots were immediately stored at −80 °C until further analysis.

### 4.4. Extraction and Quantification of cfDNA

cfDNA was extracted from plasma samples using the Maxwell^®^ RSC cfDNA LV Plasma Kit with the Maxwell^®^ RSC Instrument (Promega, Madison, WI, USA). The obtained cfDNA was immediately quantified using the Qubit dsDNA HS Assay Kit (Life Technologies, Carlsbad, CA, USA). All procedures were conducted following the manufacturer’s instructions.

### 4.5. Fragment Size Distribution Analysis

Fragment size distribution of the extracted cfDNA was assessed using a capillary electrophoresis system. Cell-free DNA ScreenTapes and reagents were used in a 4200 TapeStation instrument (Agilent Technologies Inc., Santa Clara, CA, USA) according to the manufacturer’s instructions. In accordance with established standards, cfDNA was defined as DNA fragments between 50 and 700 bp; fragments exceeding 700 bp were classified as high-molecular-weight genomic DNA contamination. The final cfDNA concentration was calculated by determining the proportion of the 50–700 bp fraction relative to the total DNA (initially quantified by Qubit) and standardized per mL of starting plasma. Mononucleosomes (MNs) were specifically defined as fragments within the 100–250 bp range.

### 4.6. Statistical Analysis

Categorical variables are presented as frequencies and percentages [*n* (%)], while continuous variables are expressed as the mean ± standard deviation (SD) for normally distributed data or as the median with interquartile range (IQR) for non-normally distributed data. Normality was assessed using the Shapiro–Wilk test.

Sample size was determined a priori using G*Power software (version 3.1.9.7). To detect a large effect size (Cohen’s d = 0.8) with a significance level of α = 0.05 and a statistical power of 90% (1-β = 0.90) using a two-tailed independent t-test, a minimum total sample size of 68 patients (34 per group) was required. Our final study cohort comprised 85 patients (39 HCC and 46 LC), ensuring that the study was sufficiently powered to identify significant differences in cfDNA parameters and diagnostic performance.

Differences between two groups were calculated with the Mann–Whitney U test for continuous variables and Pearson’s Chi-square or Fisher’s exact test for categorical variables. For comparisons involving more than two groups, the Kruskal–Wallis test followed by Dunn’s post hoc test for multiple comparisons was used. Correlations between cfDNA parameters and other variables were assessed using the Spearman rank correlation test.

Multivariate backward binary logistic regression analysis was performed to identify independent predictors for discriminating between LC and HCC patients and to develop a probability model for HCC diagnosis. Calibration of the multivariate diagnostic model was assessed using the Hosmer–Lemeshow goodness-of-fit test. To ensure the robustness and generalizability of the diagnostic model, internal validation was performed using LOOCV. Additionally, model calibration and overall performance were further validated through 1000 bootstrap resamples to calculate the Brier score and the mean absolute prediction error. Receiver Operating Characteristic (ROC) curves were used to evaluate the diagnostic utility of several parameters and the calculated model. AUROCs between variables were compared using DeLong’s test.

Prognostic factors for OS in HCC patients were investigated using the Cox Proportional Hazards Model. Survival probabilities were estimated using the Kaplan–Meier method and compared via the log-rank test. The optimal cutoff value to stratify patients into high and low groups was determined by analyzing the ROC curves for 1-year survival and selecting the point that maximizes the Youden Score, and this cutoff was subsequently used for Kaplan–Meier survival analysis to evaluate differences in survival outcomes.

Statistical significance was set at *p* < 0.05. Analyses were performed using IBM SPSS (25) and RStudio (4.4.2); graphs were created using GraphPad Prism (11.0.0) and RStudio (4.4.2).

## Figures and Tables

**Table 1:** Baseline characteristics and clinical data of patients.

Variable	LC, *N* = 46	HCC, *N* = 39	*p*-Value
Gender, *N* (%)			**<0.001**
Female	20 (43.5)	2 (5.1)	
Male	26 (56.5)	37 (94.9)	
Age (Years)	66.7 ± 9.4	66.4 ± 9.5	0.90
Child Pugh–Turcotte, *N* (%)			
A	46 (100)	30 (76.92)	
B		7 (17.95)	
C		2 (5.13)	
Cirrhosis etiology, *N* (%)			0.14
Alcohol	14 (30.4)	17 (43.6)	
HCV	19 (41.3)	16 (41.0)	
HBV	2 (4.4)	3 (7.7)	
MASLD	4 (8.7)		
Autoimmunne	4 (8.7)		
Unknown	3 (6.5)	3 (7.7)	
Diabetes mellitus, *N* (%)	14 (30.4)	13 (33.3)	0.82
Hypertension, *N* (%)	28 (60.9)	18 (46.1)	0.20
Alcohol consumption, *N* (%)	16 (34.8)	29 (74.4)	**<0.001**
Mortality, *N* (%)	3 (6.5) *	29 (74.4)	**<0.001**
AFP (ng/mL)	3.6 (2.3–4.9)	7.65 (3.18–42.6)	**<0.001**
CRP (mg/L)	2.2 (0.98–5.2)	9.9 (3.78–27.48)	**<0.001**
Albumin (g/dL)	4.1 (3.8–4.4)	3.75 (3.28–4.03)	**<0.001**
Platelets (10^6^/L)	125 (94–170)	126 (92–176)	0.83
INR	1.05 (0.98–1.11)	1.14 (1.09–1.28)	**<0.001**
Prothrombin activity (%)	89.6 (80.62–104)	78.5 (71–87)	**0.001**
AST (U/L)	47.5 (30.5–60.25)	70 (41–99)	**0.041**
ALT (U/L)	29.5 (17.75–43.5)	31 (17–45)	0.75
GGT (U/L)	44.5 (30.75–104)	122 (59–252)	**0.001**
ALP (U/L)	91 (72.5–115.25)	126 (88–157)	**0.007**
BCLC, *N* (%)			
Early: A		15 (38.5)	
Advanced: B		13 (33.3)	
C		5 (12.8)	
D		6 (15.4)	
LVI, *N* (%)		5 (12.8)	
Localization, *N* (%)			
Right		21(53.8)	
Left		8 (20.5)	
Bilobule		10 (25.6)	
Nº of nodules, *N* (%)			
1		17 (44.7)	
2–3		12 (31.6)	
>3		9 (23.7)	
Treatment, *N* (%)			
Surgical		9 (23.1)	
Transplant		6 (15.4)	
Resection		3 (7.7)	
Not surgical		30 (76.9)	
Chemoembolization		15 (38.5)	
Sorafenib		5 (12.8)	
Radio Frequency		4 (10.3)	
Symptomatic		6 (15.4)	

Qualitative data shown as *n* (%) of patients and quantitative data shown as mean ± SD or median (interquartile range). *p*-value shown for differences between LC and HCC groups calculated with Mann–Whitney U (continuous variables) or Chi-square (categorical variables) tests. Bold values indicate statistical significance (*p* < 0.05). * Causes of death in the LC group: subdural hematoma (*n* = 1), pneumonia (*n* = 1), and cardiorespiratory arrest (*n* = 1). Abbreviations: AFP, alpha-fetoprotein; BCLC, Barcelona Clinic Liver Cancer; CRP, C-reactive protein; LC, liver cirrhosis; N, number of individuals; MASLD, metabolic dysfunction-associated steatotic liver disease; INR, international normalized ratio; LVI, lymphovascular invasion.

**Table 2:** Associations between CMAC score and patient’s clinicopathological features.

Categorical Variables	*p*-Value	
Cirrhosis etiology	0.681	
Diabetes mellitus	0.587	
Hypertension	0.390	
Alcohol consumption	**0.049**	
BCLC	**0.009**	
LVI	**0.007**	
Localization	0.364	
Nº of nodules	0.370	
**Continuous Variables**	***p*** **-Value**	**Spearman Rho**
Age (years)	0.782	0.03
AFP (ng/mL) *	**<0.001**	0.682
CRP (mg/L) *	**<0.001**	0.724
Albumin (g/dL)	**<0.001**	−0.420
Platelets (10^6^/L)	0.887	0.016
INR	**0.003**	0.323
Prothrombin activity	**0.003**	−0.313
AST (U/L)	**0.005**	0.398
ALT (U/L)	0.451	0.083
GGT (U/L)	**<0.001**	0.514
ALP (U/L)	**0.010**	0.280

The distribution between groups for categorical variables, analyzed by Mann–Whitney or Kruskal–Wallis tests, and correlations for continuous variables with Spearman test. Bold values indicate statistical significance (*p* < 0.05). * AFP and CRP are components of the CMAC score. Note: Clinicopathological features exclusive to malignancy (e.g., BCLC, LVI, localization and Nº of nodules) were analyzed only within the HCC patient subgroup. Abbreviations: AFP, alpha-fetoprotein; BCLC, Barcelona Clinic Liver Cancer; CRP, C-reactive protein; INR, international normalized ratio; LVI, lymphovascular invasion.

**Table 3:** Univariate and multivariate Cox’s proportional hazard regression analysis for OS in the entire HCC cohort and in ES or AS.

	Univariate				Final Multivariate			
HCC (*N* = 39)	HR	95%CI		*p*	HR	95%CI		*p*
BCLC (early/advanced)	4.305	1.808	10.249	**0.001**	4.049	1.675	9.788	**0.002**
cfDNA concentration (ng/mL)	1.029	1.010	1.047	**0.002**	1.027	1.007	1.047	**0.008**
GGT (U/L)	1.003	1.001	1.005	**0.002**	1.003	1.000	1.005	**0.018**
Treatment (surgical/not surgical)	4.587	1.378	15.273	**0.013**				
AFP (ng/mL)	1.000	1.000	1.001	**0.034**				
CRP (mg/L)	1.117	1.006	1.241	**0.039**				
CMAC score (high/low)	3.044	1.387	9.680	**0.005**				
**ES (*N* = 15)**								
Hypertension, *N* (%)	8.877	1.053	74.826	**0.045**				
GGT (U/L)	1.006	1.000	1.012	**0.044**				
**AS (*N* = 24)**								
cfDNA concentration (ng/mL)	1.031	1.010	1.052	**0.003**	1.031	1.010	1.052	**0.004**
GGT (U/L)	1.002	1.000	1.005	**0.043**				

Bold values indicate statistical significance (*p* < 0.05). Abbreviations: AS, advanced stage; AFP, alpha-fetoprotein; BCLC, Barcelona Clinic Liver Cancer; CMAC, concentration, mononucleosome, alpha-fetoprotein and C-reactive protein; CRP, C-reactive protein; cfDNA, circulating cell-free DNA; ES, early stage; GGT, gamma-glutamyltransferase; HR, hazard ratio; N, number of individuals; OS, overall survival.

## Data Availability

The main data supporting the findings of this study are included within the article and its Supplementary Material. The raw datasets are not publicly available due to patient privacy constraints but can be made available from the corresponding author upon reasonable request

## Supplementary Materials

The following supporting information can be downloaded at: https://www.mdpi.com/article/10.3390/ijms27125590/s1.

## Abbreviations

AFP	alpha-fetoprotein
ALP	alkaline phosphatase
ALT	alanine aminotransferase
AS	advanced stage
AST	aspartate transaminase
AUC	area under the curve
AUROC	area under the Receiver Operating Characteristic
BCLC	Barcelona Clinic Liver Cancer
cfDNA	cell-free DNA
CMAC	concentration, mononucleosome, alpha-fetoprotein and C-reactive protein
CRP	C-reactive protein
ES	early stage
GGT	gamma-glutamyltransferase
HCC	hepatocellular carcinoma
HR	hazard ratio
IQR	interquartile range
INR	international normalized ratio
LC	liver cirrhosis
LOOCV	Leave-One-Out Cross-Validation
LVI	lymphovascular invasion
MASLD	metabolic dysfunction-associated steatotic liver disease
MN	mononucleosome
N	number of individuals
OS	overall survival
ROC	Receiver Operating Characteristic
SD	standard deviation
