# Supplementary material for: Circulating Cell-Free DNA Analysis for Diagnostic and Prognostic Assessment of Hepatocellular Carcinoma in Cirrhosis
Source: Int J Mol Sci. 2026 Jun 20;27(12):5590. doi: 10.3390/ijms27125590 (PMC13299745; doi:10.3390/ijms27125590)
Supplement: Supplementary file 1 [file ijms-27-05590-s001.zip › ijms-4352142-supplementary.pdf]

SUPPLEMENTARY FIGURES

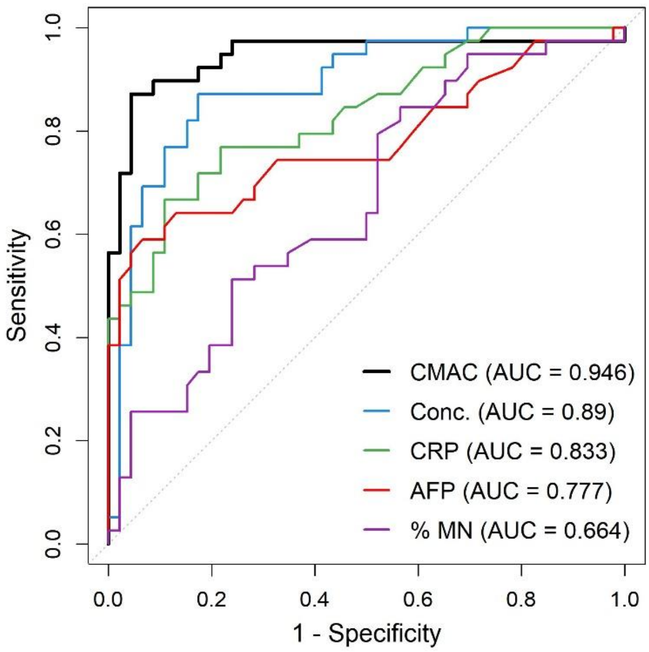

Figure S1. Diagnostic accuracy of the CMAC model compared to its individual components. ROC curves for the CMAC index and each standalone biomarker in the total study cohort. The CMAC model (black line) is shown alongside its constituent parameters: cfDNA concentration (blue), CRP (green), AFP (red), and % MN (purple). Diagonal dashed line represents the line of identity (AUC = 0.5). Abbreviations: AFP, alpha-fetoprotein; CRP, C-reactive protein; cfDNA, circulating cell-free DNA; CMAC, Concentration, MN, Alpha-fetoprotein and C-reactive protein; LC, liver cirrhosis; MN, mononucleosomes; ROC, receiver operating curve.

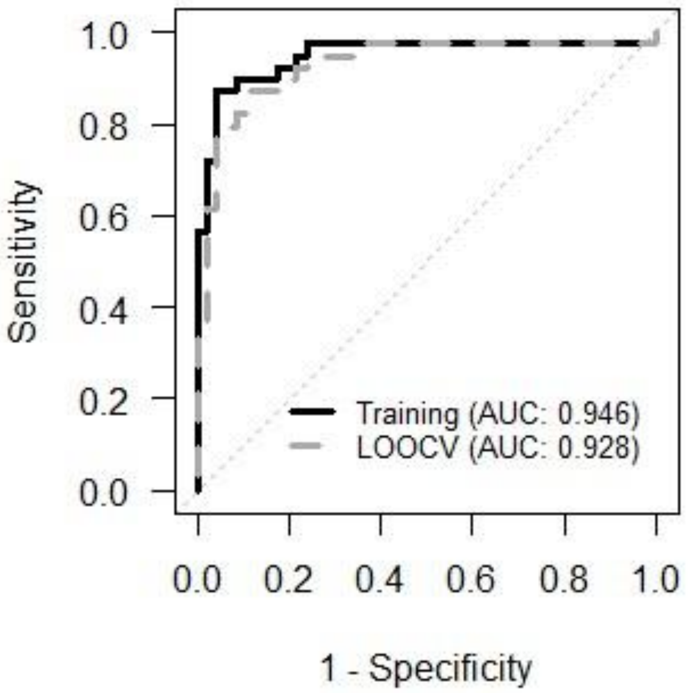

Figure S2. Internal validation of the CMAC model performance. ROC curves comparing the original training performance (black solid line) and the LOOCV performance (grey dashed line). The

minimal decrease in the area under the curve (AUC) from the training set (0.946) to the cross-validated estimate (0.928) indicates the high stability and predictive robustness of the CMAC index. Diagonal dashed line represents the line of identity (AUC = 0.5). LOOCV, Leave-One-Out Cross-Validation; ROC, receiver operating curve.

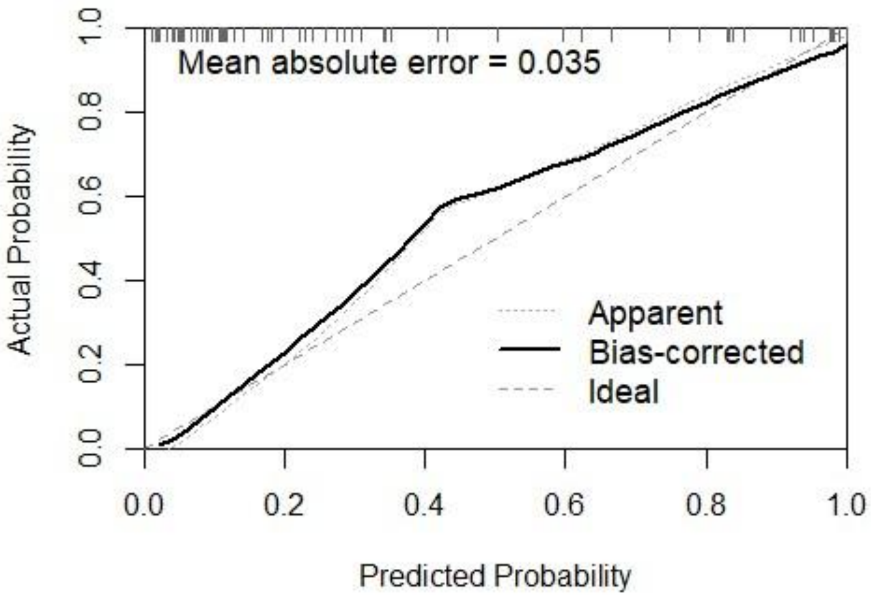

Figure S3. Calibration plot of the CMAC model. Calibration curve illustrating the relationship between the predicted probability of hepatocellular carcinoma (x-axis) and the actual observed proportion of cases (y-axis). The dashed line represents the ideal calibration (intercept = 0, slope = 1). The bias-corrected line (solid black line), calculated via bootstrapping, shows close alignment with the ideal diagonal, supported by a low mean absolute error (MAE = 0.035).

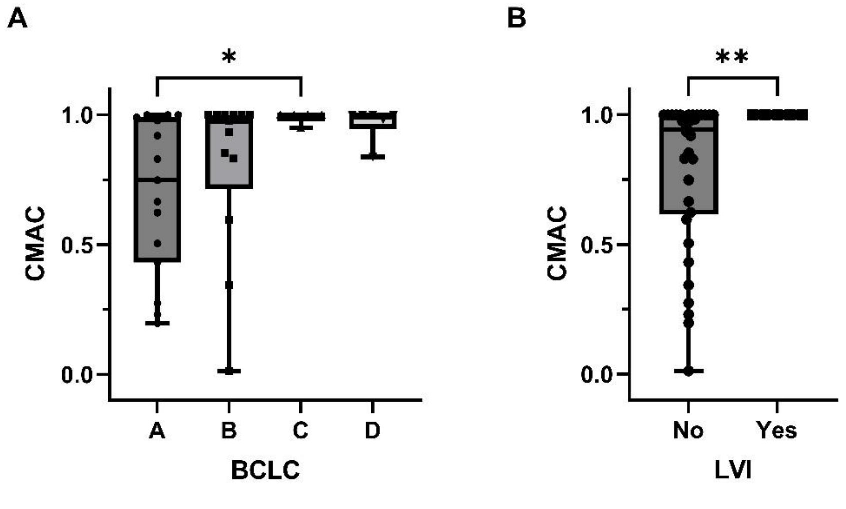

Figure S4. Correlation of the CMAC index with clinical stages and pathological features. Boxplots representing the CMAC index distribution according to (A) BCLC staging and (B) presence of LVI. *p*-values for Kruskall-Wallis and Mann-Whitney test, respectively, *$p<0.05$, **$p<0.01$. BCLC: Barcelona Clinic Liver Cancer; LVI: lymphovascular invasion.

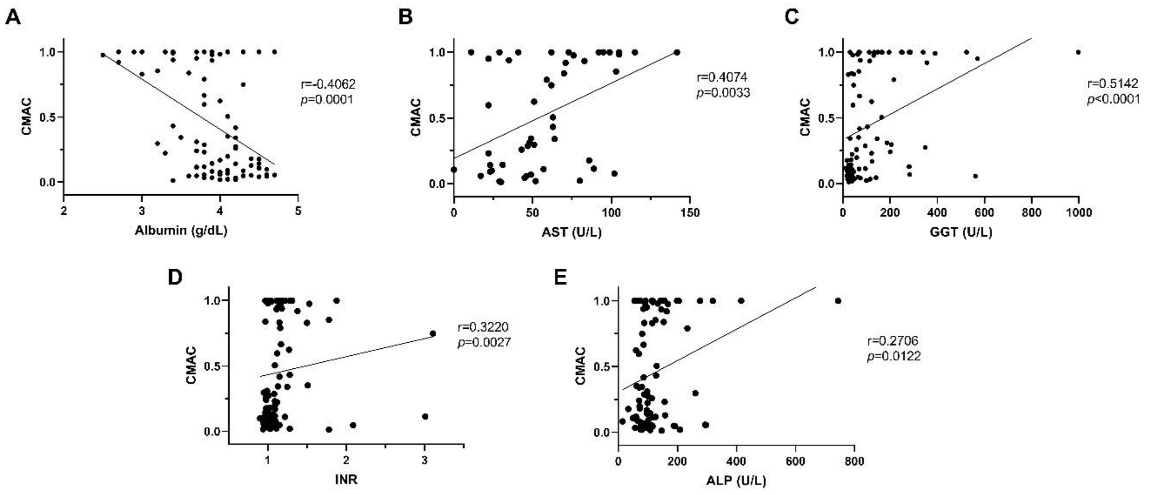

Figure S5. Correlation analysis between the CMAC index and biochemical parameters of liver function. Scatter plots illustrating the Spearman correlation between CMAC index scores and key clinical biomarkers: (A) Albumin, (B) AST, (C) GGT, (D) INR, and (E) ALP. Individual patient data are represented by dots with a linear regression line superimposed for visual representation. r: Spearman's correlation coefficient; AST: aspartate aminotransferase; GGT: gamma-glutamyl transferase; INR: international normalized ratio; ALP: alkaline phosphatase.

SUPPLEMENTARY TABLES

Table S1. Baseline characteristics and clinical data of HCC patients divided into early and advanced BCLC stages.

Variable	ES (N=15)	AS (N=24)	*p*-value
Gender, N (%)			0.73
Female	1 (6.67)	1 (4.17)	
Male	14 (93.3)	23 (95.8)	
Age (Years)	64.4+9.1	67.8+9.3	0.28
Child Pugh-Turcotte, N (%)			0.83
A	11 (73.3)	18 (75)	
B	2 (13.3)	5 (20.8)	
C	1 (6.7)	1 (4.2)	
Cirrhosis Etiology			0.29
Alcohol	8 (53.3)	9 (37.5)	
HCV	5 (33.3)	11 (45.8)	
HBV	2 (13.3)	1 (4.17)	
Unknown	0	3 (12.5)	
Diabetes mellitus, N (%)	6 (40)	7 (29.2)	0.51
Hypertension, N (%)	8 (53.3)	10 (41.7)	0.53
Alcohol consumption, N (%)	13 (86.7)	16 (66.7)	0.26

Mortality, N (%)	7 (46.7)	22 (91.7)	**0.003**
AFP (ng/mL)	5.8 (3.0-9.2)	22.00 (4.6-205.7)	**0.006**
CRP (mg/L)	6.8 (2.9-24.5)	17.75 (7.8-28.5)	0.18
Albumin (g/dL)	3.8 (3.4-4.1)	3.6 (3.2-4.0)	0.62
Platelets (10^6/L)	100 (66-151)	130.5 (104.0-198.5)	0.06
INR	1.1 (1.0-1.3)	1.15 (1.1-1.2)	0.62
Prothrombin activity (%)	82.0 (66.0-91.5)	78.35 (72.5-85.5)	0.99
AST (U/L)	63.0 (53.8-96.5)	73.00 (30.0-99.0)	0.98
ALT (U/L)	33.00 (17-40)	30.50(17.0-45.8)	0.94
GGT (U/L)	104 (46-164)	139.5 (64.3-273.8)	0.42
ALP (U/L)	115 (72-134)	143.5 (93.3-191.3)	0.05
LVI, N (%)	0	5 (20.8)	0.14
Localization, N (%)			0.15
Right	11 (73.3)	10 (41.7)	
Left	2 (13.3)	6 (25)	
Bilobule	2 (13.3)	8 (33.3)	
Nº of nodules, N (%)			0.43
1	7 (46.7)	10 (41.7)	
2-3	6 (40)	6 (25)	
>3	2 (13.3)	7 (29.2)	
Treatment, N (%)			**0.001**
Surgical	8 (53.3)	1 (4.2)	
Transplant	6 (40)	0	
Resection	2 (13.3)	1 (4.2)	
Not surgical	7 (46.7)	23 (95.8)	
Chemoembolisation	4 (26.7)	11 (45.8)	
Sorafenib	0	5 (20.8)	
Radio Frequency	3 (20)	1 (4.2)	
Symptomatic	0	6 (25)	

Qualitative date shown as n (%) of patients and quantitative data shown as as mean±SD or median (interquartile range). *p*-value shown for differences between LC and HCC groups calculated with Mann-Whitney U (continuous variables) or or Chi-square (categorical variables) tests. Bold values indicate statistical significance (p<0.05). Abbreviations: AFP, alpha-fetoprotein; BCLC, Barcelona Clinic Liver Cancer; CRP, C-reactive protein; LC, liver cirrhosis; N, number of individuals; MASLD, metabolic dysfunction-associated steatotic liver disease; INR, international normalized ratio; LVI, lymphovascular invasion.
